# Cochlear Synaptic Degeneration and Regeneration After Noise: Effects of Age and Neuronal Subgroup

**DOI:** 10.3389/fncel.2021.684706

**Published:** 2021-08-09

**Authors:** Tyler T. Hickman, Ken Hashimoto, Leslie D. Liberman, M. Charles Liberman

**Affiliations:** ^1^Eaton-Peabody Laboratories, Massachusetts Eye and Ear, Boston, MA, United States; ^2^Department of Otolaryngology–Head and Neck Surgery, Harvard Medical School, Boston, MA, United States; ^3^Department of Otolaryngology-Head and Neck Surgery, Tohoku University School of Medicine, Sendai, Japan

**Keywords:** ribbon synapse, synaptopathy, regeneration, noise overexposure, cochlea, spiral ganglion, aging, spontaneous rate

## Abstract

In CBA/CaJ mice, confocal analysis has shown that acoustic overexposure can immediately destroy synapses between auditory-nerve fibers (ANFs) and their peripheral targets, the inner hair cells (IHCs), and that years later, a corresponding number of ANF cell bodies degenerate. In guinea pig, post-exposure disappearance of pre-synaptic ribbons can be equally dramatic, however, post-exposure recovery to near-baseline counts has been reported. Since confocal counts are confounded by thresholding issues, the fall and rise of synaptic ribbon counts could represent “regeneration,” i.e., terminal retraction, re-extension and synaptogenesis, or “recovery,” i.e., down- and subsequent up-regulation of synaptic markers. To clarify, we counted pre-synaptic ribbons, assessed their juxtaposition with post-synaptic receptors, measured the extension of ANF terminals, and quantified the spatial organization and size gradients of these synaptic elements around the hair cell. Present results in guinea pigs exposed as adults (14 months), along with prior results in juveniles (1 month), suggest there is post-exposure neural regeneration in the guinea pig, but not the CBA/CaJ mouse, and that this regenerative capacity extends into adulthood. The results also show, for the first time, that the acute synaptic loss is concentrated on the modiolar side of IHCs, consistent with a selective loss of the high-threshold ANFs with low spontaneous rates. The morphological similarities between the post-exposure neurite extension and synaptogenesis, seen spontaneously in the guinea pig, and in CBA/CaJ only with forced overexpression of neurotrophins, suggest that the key difference may be in the degree of sustained or injury-induced expression of these signaling molecules in the cochlea.

## Introduction

Most hearing impairment among adult humans, including noise-induced hearing loss, involves damage to either cochlear hair cells or their afferent innervation, the auditory nerve fibers (ANFs). Ultrastructural work in cat and guinea pig in the early 1980’s (Liberman and Mulroy, 1982; Robertson, 1983) showed swelling and rupture of ANF terminals at their synaptic connections with inner hair cells (IHCs) when examined within 24 h of acoustic overexposure. This neural damage could be mimicked in guinea pigs by perfusing the cochlea with glutamate agonists, and it could be partially blocked by perfusion with glutamate antagonists, suggesting neural excitotoxicity at this highly active, glutamatergic synapse (Puel et al., 1998; Ruel et al., 2007). Furthermore, mice with genetic or pharmacologic blockade of glutamatergic neurotransmission showed reduced susceptibility to noise-induced synaptopathy (Kim et al., 2019; Hu et al., 2020).

This acute noise-induced damage was seen after exposures that were completely reversible with respect to cochlear thresholds in animals with longer post-exposure survival. Furthermore, swollen ANF terminals in cats were no longer seen a few days after overexposure, and the synaptic ultrastructure on surviving IHCs appeared normal (Liberman and Mulroy, 1982). These observations suggested that the myelinated peripheral axons of these bipolar neurons (as well as their cell bodies and central axons) survive the loss of their unmyelinated peripheral terminals and can extend new neurites to re-establish synaptic connections with their IHC targets (Ruel et al., 2007).

However, the ultrastructural studies of acute and chronic acoustic injury did not count ANF terminals and/or synapses. Thirty years later, studies in noise-damaged mice used immunostaining for pre- and post-synaptic markers along with confocal imaging to count synapses in large numbers of IHCs before and after noise exposures causing large acute, but ultimately reversible, threshold shifts (Kujawa and Liberman, 2009; Fernandez et al., 2015; Liberman L. D. et al., 2015). These confocal studies showed immediate loss of up to 50% of the synapses between IHCs and ANFs. The loss remained stable from 1 day to 8 weeks post exposure, and when animals survived for 2 years post exposure, loss of ANF cell bodies closely approached the 50% value of synaptic loss seen at day 1 (Kujawa and Liberman, 2009).

The apparent discrepancy between ultrastructural studies in guinea pig and confocal studies in CBA/CaJ mice could be explained as follows: (1) a 50% synaptic loss could have been undetected in random-section ultrastructural studies, and (2) silencing 50% of ANFs would not elevate cochlear electrophysiological thresholds by more than 10 dB (Lobarinas et al., 2013; Bourien et al., 2014), because activity spreads so rapidly along the cochlear spiral as sound pressure is increased. Thus, the acute noise-induced threshold shifts were dominated by outer hair cell (OHC) dysfunction (Darrow et al., 2007), and thresholds recovered because hair cell function recovered. Although the ANF synaptic damage was irreversible, cochlear threshold measures were insensitive to this primary neural degeneration.

Despite this reasonable resolution, a subsequent confocal study suggested that noise-damaged ANF terminals actually do regenerate in guinea pig (Shi et al., 2013). After an exposure that produces massive loss of pre-synaptic ribbons at 1 day post-exposure, ribbon counts return to near-normal levels within a few weeks. Although counting immunostained ribbons is a powerful way to sample large numbers of hair cells, the disappearance and reappearance of immunofluorescent puncta could reflect down- and up-regulation of the pre-synaptic immunomarker rather than degeneration and regeneration of ANF terminal dendrites followed by synaptogenesis. The distinction is critical, especially in the context of designing therapies to elicit neurite extension and synaptogenesis in humans, where the ANF peripheral axon has degenerated although the IHC targets survive (Liu et al., 2015; Wu et al., 2020).

We addressed this with a confocal study of noise-induced synaptopathy in guinea pig by adding immunomarkers for post-synaptic receptors and ANF terminals, and by analyzing the locations and sizes of synaptic complexes around the IHC (Hickman et al., 2020). By showing that the post-exposure repopulation of IHCs with pre-synaptic puncta is accompanied by the appearance of neurites and post-synaptic receptor patches far from their normal locations, we provided evidence for synaptic plasticity as well as post-exposure neurite extension and synaptogenesis in this noise damage model.

However, several key questions remained, including the role of age-at-exposure. Noise vulnerability in mice decreases as animals mature from pre- to post-pubescent (Bock and Saunders, 1977; Henry, 1983; Kujawa and Liberman, 2006), and cochlear regenerative capacity decreases with postnatal age, at least within the first few weeks (Martinez-Monedero et al., 2007; Cox et al., 2014). These age-at-exposure differences can be dramatic: e.g., 40 dB more permanent threshold shift in animals exposed at 6 weeks vs. 16 weeks (Kujawa and Liberman, 2006). Prior guinea pig studies by our group (Hickman et al., 2020) and others (Shi et al., 2013), noise-exposed animals as juveniles, around the onset of puberty, whereas the original mouse work on cochlear synaptopathy (Kujawa and Liberman, 2009) exposed mice as mature post-pubescents (∼16 weeks). The mouse data is further complicated by inter-strain differences. The original work (showing no regenerative capacity) was done in CBA/CaJ, whereas more recent studies in C57BL/6 mice report substantial recovery of ribbon counts in animals exposed at 5–6 weeks (Shi et al., 2015; Kaur et al., 2019) and more limited recovery in ear exposed at 8–12 weeks (Kim et al., 2019). Intriguingly, one of the C57BL/6 studies reports that, while ribbons recovered from an exposure at 6 weeks, they do not after a second exposure at 10 weeks (Luo et al., 2020). Although the role of age vs. repeat-exposure in generating this difference was not explored, the results emphasize the need to clarify the effects of age-at-exposure on the regenerative capacity of the inner ear, which was one aim of the present study.

Another aim was to resolve the discrepancy between physiological and morphological evidence as to the differential vulnerability among ANF functional subgroups. Our 2013 neurophysiological study of single ANFs in synaptopathic guinea pigs (Furman et al., 2013) suggested that ANFs silenced by synaptic loss were predominately those with high thresholds and low spontaneous rates (SRs). However, low-SR ANFs make synaptic contacts exclusively on the “modiolar” side of the IHC (Liberman, 1982), and our prior neurophysiological study of synaptopathic guinea pigs saw no difference in the number of ribbons lost from the two sides of the IHC (Furman et al., 2013). However, both the neurophysiological and histopathological analyses of these noise-exposed guinea pigs were carried out at 3 – 5 wks post exposure and therefore must have been complicated by the ongoing synaptic re-organization during this active regenerative phase. Thus, the present study also evaluated the synaptic architecture acutely post-exposure to look for more clear-cut differences between the damage on the two sides of the IHCs.

## Materials and Methods

### Subjects and Groups

Female Hartley guinea pigs, received from the animal supplier (Elm-Hill Labs) at ages of either 1 month (juveniles) or 14 months (mature), were used in this study. Data from the juveniles included a mix of new subjects and subjects from a prior report (Hickman et al., 2020). After an acclimation of 2 weeks to the animal care facility, animals were placed awake and unrestrained in a wire-mesh cage on a rotating platform within a reverberant chamber and exposed to noise (4–8 kHz, 106 dB SPL, 2 h), designed to induce large threshold shifts with minimal hair cell loss. Animals were allowed to recover for 1 day (mature and juvenile); 1, 4, or 8 weeks (juvenile); or 6 weeks (mature) before cochlear function tests, i.e., CAPs (compound action potentials) and DPOAEs (distortion product otoacoustic emissions), immediately followed by tissue harvest. Mice from the CBA/CaJ strain were exposed awake and unrestrained for 2 h to an octave-band noise (8–16 kHz) at either 8 or 16 weeks of age, at 98 or 100 dB SPL, respectively, and allowed to recover for varying periods before cochlear function tests (DPOAEs and either CAPs or ABRs) immediately followed by tissue harvest.

**FIGURE 1:**
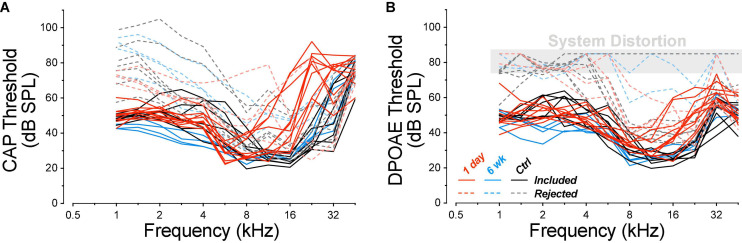
Animals with exceptionally high DPOAE thresholds at low frequencies were excluded from the study. Thresholds for CAPs **(A)** and DPOAEs **(B)** for all the animals tested are coded for experimental group, as indicated in the key, and separated into two non-overlapping groups (dashed vs. solid curves) according to their DPOAE thresholds near 2 kHz. DPOAE thresholds at and above 75 dB SPL are indicated as “system distortion,” because DPOAEs were generated in a passive coupler when the intensity of the primaries reached this level. Group sizes before case exclusion were: control *n* = 14 ears of 7 animals, 1 day *n* = 14 ears of 7 animals, 6 weeks *n* = 8 ears of 4 animals.

At the time of cochlear function testing, roughly half of the mature guinea pigs, both control and exposed, showed abnormally high CAP thresholds at frequencies ≤2 kHz, and DPOAE thresholds at such high levels that the responses were indistinguishable from system distortion (Figure 1). Because such a pattern was never observed in the juveniles, and because this pattern strongly suggested the presence of conductive hearing loss, the high-threshold animals were removed from the study. In the final mature dataset, the number of ears were seven controls, 10 at 1-day post exposure, and four at 6-week post exposure. In the final juvenile dataset, the number of ears was 13 in the control group and 6 each in the 1-day, 1-week, 4-week, and 8-week groups, respectively. Unexposed controls were housed with exposed animals of the same age. All procedures were approved by the IACUC of the Massachusetts Eye and Ear and were performed in accordance with the “Guide for Care and Use of Laboratory Animals.”

### Cochlear Function

Cochlear function tests were carried out in an electrically shielded and sound-attenuating chamber, maintained at 32°C. Animals were deeply anesthetized with ketamine (100 mg/kg) and xylazine (8 mg/kg). Acoustic stimuli were delivered through a closed acoustic system, consisting of two sound sources (CDMG15008- 03A, CUI) and an electret condenser microphone (FG-23329-PO7, Knowles) as an in-dwelling probe microphone. Stimuli for CAPs were 4-msec tone pips (0.5 ms rise-fall, 19/s repetition rate). CAP responses were recorded with a silver-ball electrode on the round window, referred to a ground in the neck muscle near the vertex. Threshold was defined as the sound level, at each frequency, required to produce a response of 10 μV peak to peak, as determined by a computer-driven algorithm. DPOAEs were recorded in response to a pair of pure tones (*f*_1_ and *f*_2_), in a frequency ratio (*f*_2_/*f*_1_) of 1.2, and a level separation of 10 dB (*f*_1_ level > *f*_2_ level). DPOAE threshold was defined as the level of *f*_2_ required to produce a distortion product in the ear canal of 0 dB SPL, as determined by interpolation between 5 dB level steps at each *f*_2_ frequency.

### Tissue Fixation, Immunostaining and Image Acquisition

Animals were deeply anesthetized with fatal-plus solution (300 mg/kg pentobarbital sodium), and then tissues were fixed by intravascular perfusion of 4% paraformaldehyde following a saline wash-out; cochleae were flushed through the scalae with the same fix and then post-fixed for 2 h, decalcified for 2–3 weeks in 0.12 M EDTA, and dissected into pieces, each containing a fraction of the sensory epithelium. Tissue was permeabilized by freezing on dry ice in 30% sucrose and blocked for 1 h at 22°C in PBS with 1% Triton X + 5% normal horse serum. Tissue was then incubated overnight at 37°C in the following primary antibodies, diluted in 1% normal horse serum and 1% Triton X in PBS: (1) mouse isotype IgG1 anti-C-terminal binding protein 2 (CtBP2, 1:50, BD Transduction Laboratories #612044), (2) mouse isotype IgG2a anti-glutamate receptor 2 (GluA2, 1:1000, Millipore #MAB397), (3) rabbit anti-myosin VIIa (Myo7a, 1:100, Proteus BioSciences #25–6790), and (4) mouse anti-neurofilament H (NFH, 1:500, Millipore #AB5539). After washing in PBS, the tissue was incubated twice for 1 h each in the following secondaries, diluted in 1% normal horse serum and 1% Triton X in PBS: (1) Alexa Fluor 568 goat anti-mouse IgG1 (1:1000, Thermo Fisher #A21124), (2) Alexa Fluor 488 goat anti-mouse IgG2a (1:500, Thermo Fisher #A21131), (3) Pacific Blue goat anti-rabbit (1:200, Thermo Fisher P10994), and (4) Alexa Fluor 647 goat anti-chicken (1:200, Thermo Fisher #A21449).

Low-power images of the myosin channel in each microdissected piece were obtained with a 10x objective (N.A. 0.4) on a Leica DM5500 epifluorescence microscope at sufficient magnification to count hair cells. Cytocochleograms were constructed from these images by tracing the cochlear spiral and superimposing hash marks at each 2% length increment using a custom ImageJ plugin (Hickman et al., 2018). Frequency correlates were assigned based on the cochlear frequency map for guinea pig (Tsuji and Liberman, 1997) or mouse (Taberner and Liberman, 2005), and the organ of Corti was imaged at half- to one-octave intervals with a Leica confocal microscope using a 63× glycerol immersion objective (N.A. 1.3). At each of the desired locations (11 cochlear regions at log-spaced intervals from 0.5 to 45.2 kHz for guinea pig, and 8 cochlear regions from 5.6 to 64 kHz in mouse), in each case, two adjacent microscopic fields (∼10 IHCs per field) were imaged with a 4-channel z-stack spanning the height of the hair cells to faithfully capture all synaptic puncta in that region of interest (1024 pixels × 512 pixels in *x* and *y*, at roughly 80 nm per pixel with *z*- spacing at 0.33 μm per slice).

### Image Analysis

The numbers and volumes of ribbons were analyzed with Amira software (v 6.4, Visage Imaging) using the *connected components* feature to automate the identification of puncta in three-dimensional space, and the notation of their locations, sizes and numbers. The pairing of pre-synaptic ribbons with post-synaptic receptors and/or auditory nerve terminals was assessed with custom C++ software that re-projected the voxel space immediately around each ribbon (i.e., within 1 micron) as an array of thumbnail images, from which an observer can evaluate the post-synaptic staining around each ribbon: see Figure 3 in a prior report (Liberman et al., 2011) for further details. The *x*-, *y*-, *z*- coordinates of ribbons in each z-stack were transformed into a coordinate system based on the modiolar-pillar polarity of the IHCs, using a custom LabVIEW program. This program required user input only to define the line bisecting the subnuclear portion of the IHCs into pillar vs. modiolar halves, as seen in the maximum *zy* projection of each z-stack. This bisector became the transformed *y*-axis; an *x*-axis was created perpendicular to it, and was placed such that the ribbons farthest from the cuticular plate were at *y* = 0. This manual operation was performed by an observer blinded to the exposure-group/survival-time information on each ear.

### Statistics

Statistical analyses were made using Graphpad Prism v8. Each animal was considered a replicate for statistical purposes. For intergroup comparisons of metrics obtained at multiple frequencies or cochlear regions, 2-way ANOVAs were adjusted for missing values with Prism’s mixed-effects-model that calculates a compound symmetry covariance matrix, fitted using restricted maximum likelihood. This method accounts for rare, random missing values. Within the mixed-effects-model, fixed effects were defined as frequency and recovery time, while random effects were defined as subjects (animals) and residuals. The Geisser–Greenhouse correction method was used to account for unequal variance between repeated measures, and D’Agostino–Pearson’s omnibus K2 normality tests were used to verify the normality of the data. 2-way ANOVA *post hoc* multiple comparisons were calculated using the Holm–Sidak multiple comparisons method. When comparing across three groups, a Brown–Forsythe and Welch ANOVA test was performed without assuming equal standard deviations, and *post hoc* multiple comparisons were made using Dunnett’s T3 multiple comparisons test. A non-parametric two-tailed Mann-Whitney test was used to calculate pairwise comparisons.

### Data Availability

The datasets generated and analyzed in this study are available from the corresponding author on request.

## Results

### Threshold Shifts and Hair Cell Loss

**FIGURE 2:**
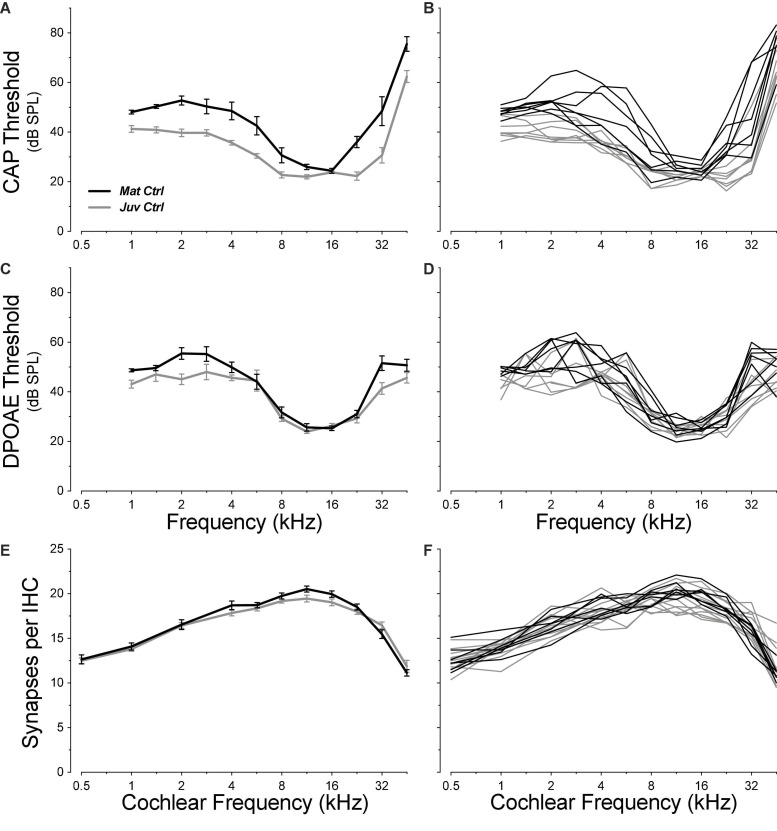
Pre-exposure thresholds were slightly higher in mature animals than in juveniles **(A–D)**, but IHC synaptic counts were statistically indistinguishable (**E,F**; group *p* = 0.48). Threshold data are shown for CAPs **(A,B)** and for DPOAEs **(C,D)**. Each metric is plotted for individual animals **(B,D,F)** and as group means ± SEMs **(A,C,E)**. Group sizes for **(A–D)** were: mature control *n* = 7 ears of 4 animals, juvenile control *n* = 8 ears of 4 animals. Group sizes for **(E–F)** were: mature control *n* = 7 ears of 4 animals, juvenile control *n* = 13 ears of 7 animals. Juvenile synapse data **(E–F)** are from a prior study (Hickman et al., 2020).

Cochlear function was assessed by recording CAPs via a round-window electrode and DPOAEs via an ear-canal sound-pressure monitor. As summarized in Figure 2, the absolute thresholds in control ears showed the expected U-shaped pattern, with maximum sensitivity between 8 and 22.6 kHz, depending on group and test. Both DPOAE and/or CAP threshold functions showed small (mean ≤ 10 dB), but significant threshold elevations in the mature ears (aged 1.3 years), at low (≤4 kHz, CAP: group *p* = 0.0055, DPOAE group: *p* = 0.0003) and high (>4 kHz, CAP group: *p* = 0.040, DPOAE group *p* = 0.34) frequencies, compared to similar measures from juveniles (aged ∼1 month).

**FIGURE 3:**
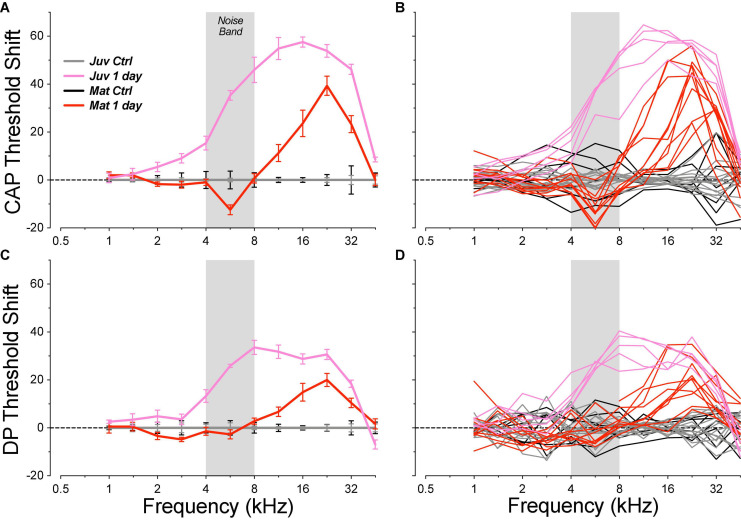
Acute threshold shifts, measured 1 day post exposure, were smaller in mature guinea pigs than in the juvenile animals from a prior report (Hickman et al., 2020). Threshold shifts are measured *re* mean control data for the same age group (see Figure 2). Data are shown for CAPs **(A,B)** and for DPOAEs **(C,D)**, and each metric is plotted for individual animals **(B,D)** and as group means ± SEMs **(A,C)**. The gray bar indicates the passband of the noise exposure used to create synaptopathy. Group sizes were: mature control *n* = 7 ears of 4 animals, juvenile control *n* = 14 ears of 7 animals, mature 1 day *n* = 10 ears of 5 animals, juvenile 1 day *n* = 5 ears of 3 animals.

As in prior studies, the noise exposure we used to induce cochlear synaptopathy was designed to produce a severe, but ultimately reversible, elevation of cochlear thresholds in the basal half of the cochlea. When measured 1 day post exposure, this 4–8 kHz octave-band noise produced peak threshold elevations near 22.6 kHz ranging, across animals, from 18 to 49 dB, by CAP, and from 8 to 35 dB when measured by DPOAEs (Figure 3). The interanimal differences in severity of threshold elevation scaled similarly in both CAP and DPOAE measures (data not shown).

The magnitude of the acute threshold elevations was significantly smaller in the mature animals than those seen after identical exposures in juveniles (Figure 3; CAP group: *p* = 0.0018, group × frequency: *p* < 0.0001; DPOAE group: *p* = 0.0006, group × frequency: *p* < 0.0001). The threshold shifts in the juveniles peaked at a lower frequency (16 vs. 22 kHz) and signs of acute damage spread to significantly lower frequency regions (2 kHz vs. 8 kHz). Enhanced noise vulnerability in immature animals has also been reported in mice (Kujawa and Liberman, 2006).

**FIGURE 4:**
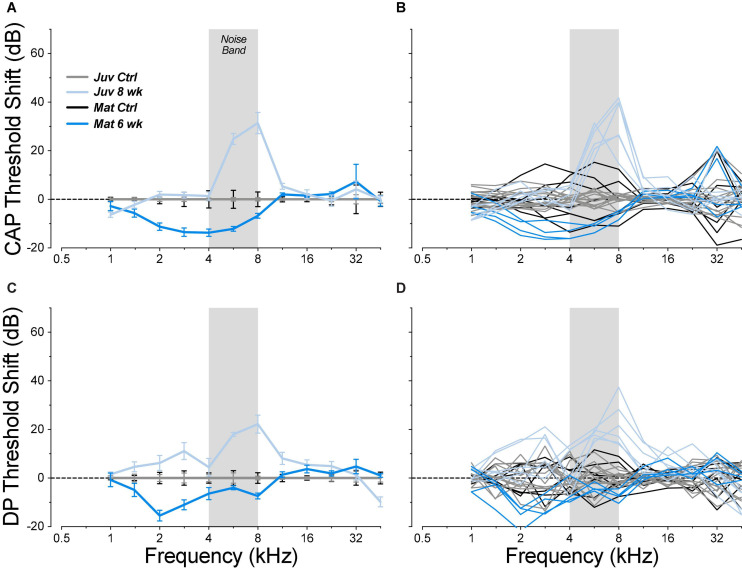
Chronic threshold shifts, measured 6 weeks post exposure, were smaller in the mature guinea pigs than in juveniles from a prior report (Hickman et al., 2020). Threshold shifts are measured *re* the mean control data for the same age group (see Figure 2). Data are shown for CAPs **(A,B)** and for DPOAEs **(C,D)**, and each metric is plotted for individual animals **(B,D)** and as group means ± SEMs **(A,C)**. The gray bar indicates the passband of the noise exposure used to create synaptopathy. Group sizes were: mature control *n* = 7 ears of 4 animals, juvenile control *n* = 14 ears of 7 animals, mature 6 weeks *n* = 4 ears of 2 animals, juvenile 8 weeks *n* = 6 ears of 3 animals.

When mature animals were allowed to survive for 6 weeks post exposure, thresholds had returned to control values at all frequencies (Figure 4), even at 22.6 kHz where the acute threshold shifts were maximal. The apparent improvement of low-frequency thresholds arises because each exposed group is compared to its age-matched controls. In fact, the mean low-frequency thresholds (1–8 kHz) in the exposed-recovered mature animals are nearly identical to those in juvenile controls (mean difference = 1 dB). We suspect that this reflects a subtle manifestation of the low-frequency threshold issues we encountered in these mature ears (Figure 1).

**FIGURE 5:**
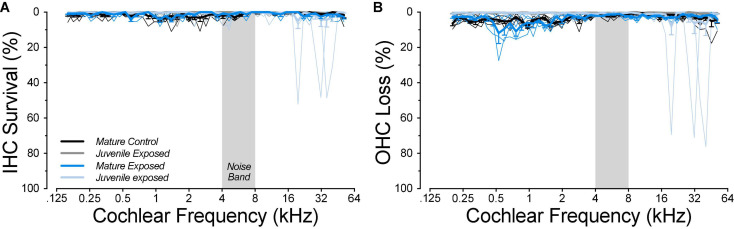
Mature controls show more baseline OHC loss than juveniles in the apical cochlea **(B)**, but, in the mature ears, noise exposure did not cause any further loss of either IHCs **(A)** or OHCs **(B)**. Data are shown both as mean values (±SEMs; thick lines), and individual cases (thin lines): both are binned in 2% increments of cochlear location. Data from juvenile animals are from a prior study (Hickman et al., 2020). Group sizes were: mature control *n* = 5 ears of 4 animals, juvenile control *n* = 6 ears of 5 animals, mature 6 weeks *n* = 4 ears of 2 animals, juvenile 1 weeks *n* = 6 ears of 5 animals, juvenile 4 weeks *n* = 6 ears of 3 animals.

As expected, based on the complete threshold recovery in the affected high-frequency cochlear regions, there was no significant loss of either IHCs or OHCs in the exposed ears (Figure 5). This differs from the noise-induced hair cell loss in the juvenile animals, where 25% (3/12) of the ears showed focal losses >48% of IHCs and ≥70% OHCs in the high-frequency regions, a phenomenon never observed in unexposed controls (Figure 5). Unexpectedly, there was a small (4.1%) but significant (Mann–Whitney *p* = 0.0006) increase in OHC loss in the apical half of the cochlea in the unexposed mature ears *re* the juvenile controls, even though our mature animals, at 1.3 years, had only aged to 25% of the 5-year mean lifespan for the laboratory guinea pig (Quesenberry and Donnelly, 2005).

### Loss and Recovery of IHC Synapses

Each auditory nerve fiber (ANF) contacts a single IHC via a single terminal swelling, forming a single synaptic plaque consisting of apposed pre- and post-synaptic active zones along with one, or (rarely) two closely spaced pre-synaptic ribbon(s) (Liberman, 1980, 1982; Liberman et al., 1990, 2011). Thus, accurate counts of the number of ANFs contacting each IHC can be obtained by immunostaining for CtBP2, a major protein in the pre-synaptic ribbon (Schmitz, 2009), and GluA2, a major AMPA receptor subunit in the post-synaptic active zone (Matsubara et al., 1996).

**FIGURE 6:**
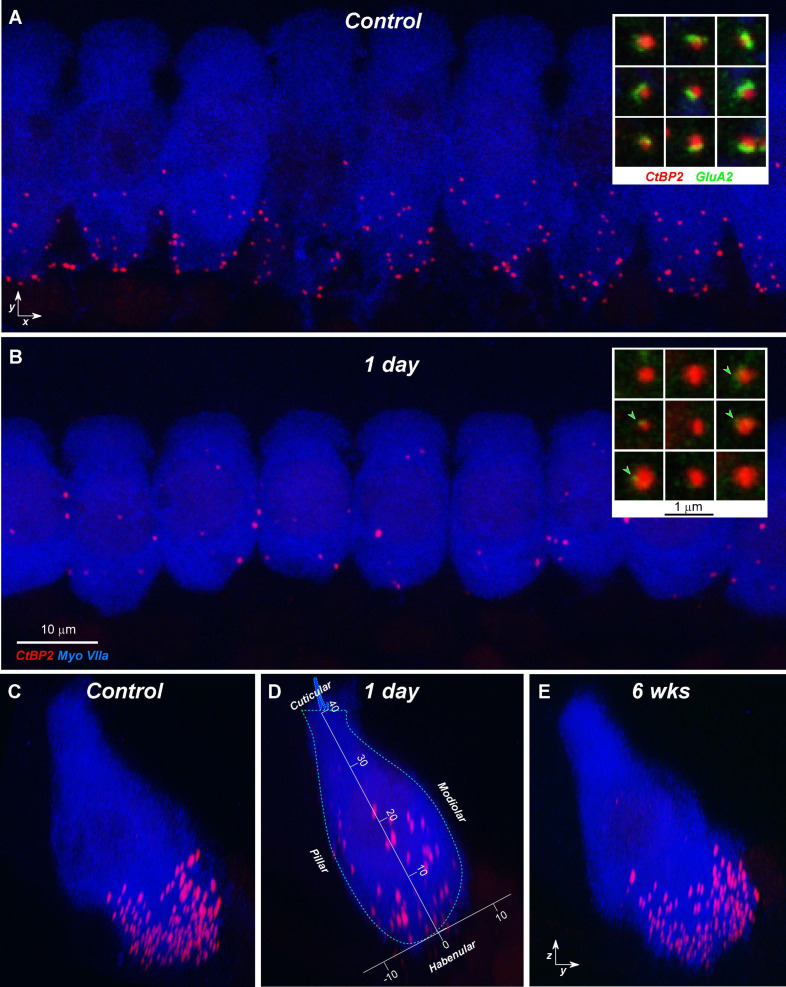
Confocal projections show the loss and recovery of synaptic ribbons after noise exposure. **(A,B)** Maximum projections from the 22.6 Hz area of nine adjacent IHCs from a control ear **(A)** and an exposed ear at 1 day post noise **(B)**. Only the CtBP2 and myosin 7a channels are shown, for clarity. Insets in each panel show nine selected ribbons from the respective z-stack, with only CtBP2 and GluA2 channels visible: arrowheads in **(B)** show tiny remnants of GluA2 signal in the 1-day post-exposure ear. **(C–E)** Reprojections of the z-stacks into the *zy* plane. Panels **(C,D)** are reprojections of **(A,B),** respectively; **(E)** is from an ear 6 weeks post noise. Panel **(D)** also shows the axis transformation used to superimpose data on ribbon position and size across multiple cases (see Figure 8). Scale bar in **(B)** also applies to **(A)**; axis scales in **(D)** are in microns and also apply to **(C,E)**.

The CtBP2 staining is typically robust enough (Figure 6) that ribbon counts can be mostly automated. The assessment of pairing between pre- and post-synaptic puncta is then facilitated by post-processing software that re-projects the 3D voxel space around each ribbon (e.g., insets to Figures 6A,B) and displays the ribbon set from each confocal stack as a thumbnail array, that can be ordered, for example, according to the overall intensity of the signal in the GluA2 channel (Liberman et al., 2011). Visual inspection of these thumbnails reveals any “orphan” ribbons, i.e., those unpaired with post-synaptic puncta: compare insets in Figures 6A,B.

**FIGURE 7:**
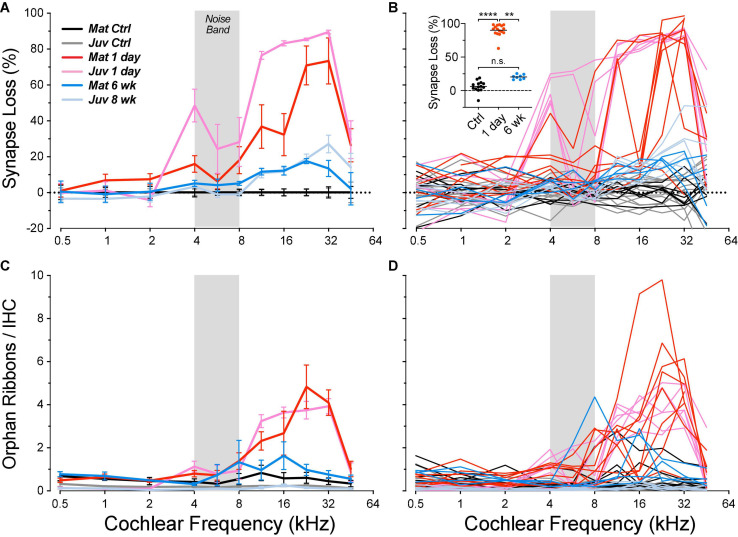
Post-exposure synapse loss and recovery are similar in juvenile and mature ears, although the acute loss is smaller in the mature cases. Panels **(A,B)** show loss of synapses, i.e., closely apposed CtBP2/GluA2 puncta, expressed *re* mean values from the appropriate unexposed control group. Panels **(C,D)** show orphan ribbon counts per IHC, i.e., CtBP2 puncta unpaired with a post-synaptic GluA2 patch. Data are shown for individual animals **(B,D)** and as group means ± SEMs **(A,C)**. Data from juvenile animals are from a prior study (Hickman et al., 2020). Group sizes were: mature control, *n* = 7 ears of 4 animals; juvenile control, *n* = 13 ears of 7 animals; mature 1 day, *n* = 8 ears of 5 animals; juvenile 1 day, *n* = 6 ears of 3 animals; mature 6 week, *n* = 4 ears of 2 animals; and juvenile 8 weeks, *n* = 6 ears of 3 animals. The inset to **(B)** shows the synaptic loss for each z-stack sampled in each mature ear at its region of peak loss. ****p < 0.0001 and **p = 0.003 by Dunnett’s T3 multiple comparisons test, comparing peak synaptopathy per animal.

In the normal guinea pig cochlea, the number of synapses, i.e., closely paired CtBP2/GluA2-positive puncta, varies between 10 and 20 per IHC, depending on cochlear location, peaking at the region tuned to 11.3 kHz (Figures 2E,F). When examined 1 day after noise exposure, there is dramatic loss of synapses in the basal half of the cochlea. Normalizing to the control counts shows that the synapse loss peaks, on average, in the region between the 22.6 and 32-kHz loci (Figure 7A). Examination of the individual-case traces shows that the loss is sometimes restricted to regions close to the 22.6-kHz place, whereas in other cases the loss extends as far apical as the 4-kHz place, i.e., to the low-frequency edge of the exposure band (Figure 7B). The acute loss of ribbons is less than the loss of synapses, because 1 day post exposure there are numerous orphan ribbons (Figures 7C,D), a phenomenon that is extremely uncommon in juvenile or mature control ears. For example, in the case illustrated in Figures 6B,D, none of the surviving ribbons was paired with robust GluA2 immunoreactivity, and the set of ribbons selected for the inset comprises those with the strongest GluA2 signal.

In other animals allowed to survive for 6 weeks post exposure, there is a dramatic recovery in the synapse counts, e.g., from a mean loss at 32 kHz of 73% on day 1 to a mean loss of only 13% at 6 weeks. The pattern and, to a lesser extent, degree of the synaptic loss and recovery in the present study of mature guinea pigs is similar to that seen in our prior study of juveniles (Hickman et al., 2020), as can be seen in Figure 7.

Whereas, in the juveniles, the acute synaptic loss was similar (80–90%) across basal-turn sampling regions from 11.3 to 32 kHz, the loss in mature ears was patchy: each ear showed peak loss near 90%, but the peaks were at different basal-turn locations. The peak values from each case are plotted, by group, in the inset to Figure 7B: the differences between control and acute (1 day) and between acute and chronic (6 weeks) are each highly significant (*p* < 0.0001 and *p* = 0.0028, respectively, by Dunnett’s T3 multiple comparisons test), whereas the differences between control and chronic are not (*p* = 0.14), indicating virtually full recovery of synaptic counts.

### Noise-Induced Changes in Synaptic Morphology and Spatial Organization

**FIGURE 8:**
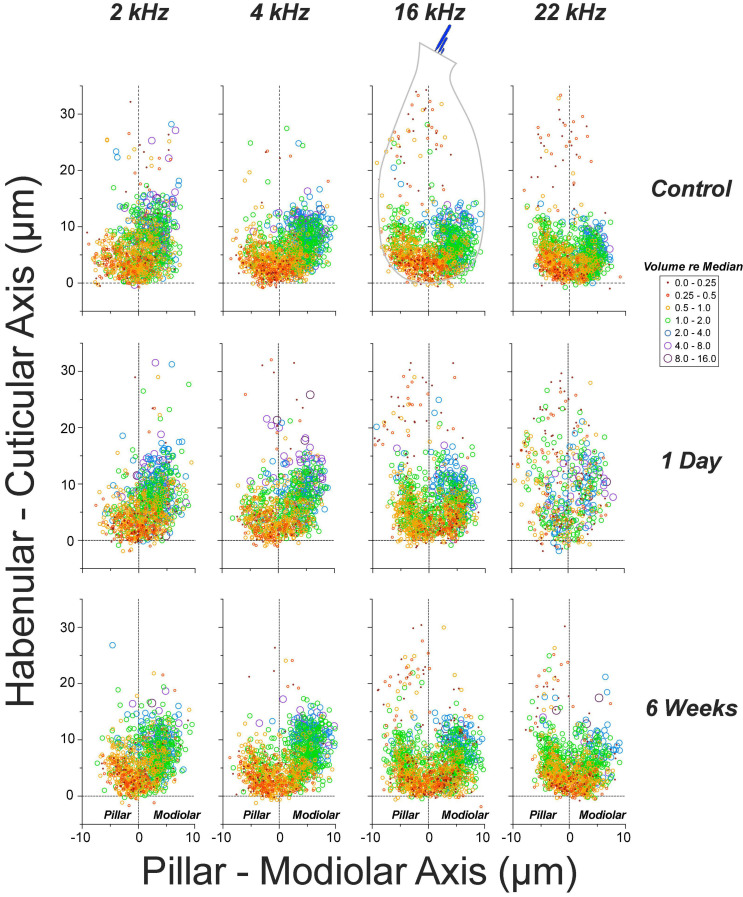
The normal ribbon-size gradient in IHCs is severely disrupted 1 day post noise, but has largely recovered 6 weeks later. Panels are organized into columns by cochlear frequency, and into rows by post-exposure time, as indicated. The locations of synaptic ribbons are shown along hair-cell-relative axes (see Figure 6D and dashed outline of 16 kHz controls for orientation): ribbon size is indicated by color and symbol size, as indicated in the key: size is normalized to the median value in each confocal z-stack. Each panel includes data from 2 image stacks (averaging 9.7 IHCs per stack – see Figures 6A,B) at each cochlear location from each ear. Data from 4 ears from 4 animals in control and 1-day group, and 4 ears from 2 animals in the 6-weeks-post-exposure group.

Single-fiber labeling experiments showed, decades ago, that ANF synapses are morphologically polarized around the IHC circumference such that most fibers with low thresholds and high spontaneous rates (SRs) are found on the side of the IHC closer to the pillar cells, whereas most of those with low SR and high thresholds are found opposite, on the modiolar side (Liberman, 1982; Tsuji and Liberman, 1997). This morphological polarization is easily seen in *yz* projections of confocal z-stacks (e.g., Figures 6C,E), because the pre-synaptic ribbons associated with low-SR (modiolar) synapses are larger than high-SR pillar-side synapses (Merchan-Perez and Liberman, 1996). The ribbon-size gradient is also obvious in the quantitative analyses in Figure 8, where we have pooled ribbon-size data from a large number of cases by defining a coordinate system aligned to the IHC row in each z-stack (Figure 6D). The synaptic puncta in the control ears are almost exclusively restricted to the basalmost 1/4 of the flask-shaped IHCs, and there is a clear gradient at all CF regions wherein larger ribbons are found on the modiolar side and smaller ribbons toward the pillar side of the IHCs.

**FIGURE 9:**
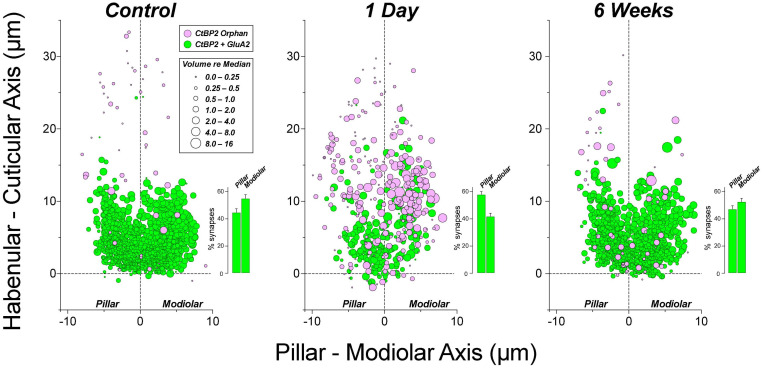
At 1 day post-exposure, many of the ribbons abnormally distant from the IHC habenular pole are unpaired with post-synaptic GluA2 patches, especially on the modiolar side of the IHC. Insets in each panel show the mean percentage of synapses (±SEMs) on the pillar vs. modiolar sides of the IHCs across all cases from the relevant group. Data are from 4 ears from 4 animals in control and 1-day groups, and 4 ears from 2 animals in the 6-weeks group. Two ears in the 1 day post exposure group that showed little synaptopathy at 22 kHz were identified as outliers (ROUT method Q = 1%), and were not included in this figure, but were included in all other figures displaying 1-day data.

The pooled data in Figure 8 also show the noise-induced disruption of the spatial organization of the ribbons and of the ribbon-size gradient at 1 day post exposure, especially at 22.6 kHz, the peak damage region, and the re-establishment of normal ribbon locations and a more normal gradient 6 weeks later. One day post exposure, many pre-synaptic puncta appear farther from the basolateral tip of the IHCs than in normal ears. As suggested by the confocal images in Figure 6, and as shown more rigorously in Figure 9, many of those migrated ribbons are orphans, i.e., no longer paired with post-synaptic GluA2 patches. This migration reverses, and the post-synaptic puncta reappear as the ear recovers. These patterns are similar to those seen in juvenile guinea pigs (Hickman et al., 2020).

**FIGURE 10:**
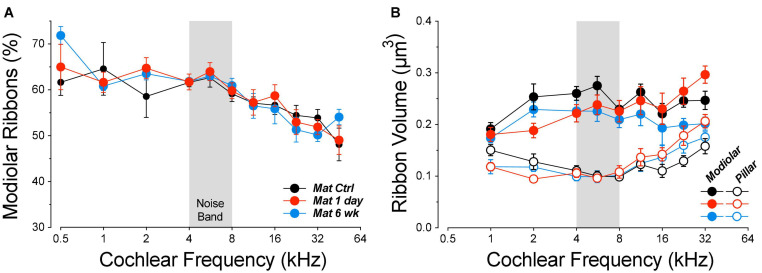
The modiolar-pillar ratio of ribbon counts is unaffected by noise exposure **(A)**, but the ribbon gradient is reduced 6 weeks after noise exposure **(B)**. Data are means (±SEMs) as extracted from the following group sizes: control *n* = 7 ears of 4 animals, 1 day *n* = 8 ears of 5 animals, 6 weeks *n* = 4 ears of 2 animals.

The synaptic disruption is more intense on the modiolar side of the IHC (Figure 9), as expected if the noise-induced damage is biased toward ANFs with low SRs (Furman et al., 2013). Although the percentage of *ribbons* on modiolar vs. pillar sides is unaffected by exposure at either 1 day or 6 weeks post (Figure 10A), the percentage of modiolar *synapses* is reduced at 1 day post exposure, as seen in the histogram insets to each panel in Figure 9. These differences in modiolar-pillar synapse distributions between both control and 6 weeks post exposure vs. 1 day post exposure are each statistically significant (*p* ≤ 0.046 by Dunnett’s T3 multiple comparisons test), whereas those between control and 6 weeks post are not (*p* = 0.83). The changes are consistent with the idea that the synaptopathy is focused primarily on post-synaptic elements, and particularly on the terminals of low-SR ANFs.

The decreasing percentage of modiolar ribbons with increasing cochlear frequency in all groups (Figure 10A) reflects the “clockwise” migration of the synaptic-contact cloud around the IHC basolateral pole, visible in the frequency-based progression of ensemble *zy* projections in Figure 8. Alterations in ribbon sizes (Figure 10B) suggest there are synaptic pathologies at locations apical to the regions with obvious ribbon loss. At 6 weeks post exposure, mean size is reduced by 15% on the modiolar side for frequencies ≤5.6 kHz (group *p* = 0.21). The size increase at high frequencies on the pillar side further weakens the normal modiolar-pillar size gradient in the frankly synaptopathic region (>8 kHz) and shows that morphological recovery of synapses is incomplete at 6 weeks. At 1 day post exposure, ribbons show a frequency-dependent size reduction (apex) and elevation (base) on both sides of the IHCs (Figure 10B, frequency × group *p* ≤ 0.0047).

## Discussion

### Synaptic Loss and Recovery in Different Mammalian Models

The phenomenon of cochlear synaptopathy, i.e., the degeneration of ANF synaptic contacts on surviving IHCs, has now been documented in numerous mammalian species, including mouse (Kujawa and Liberman, 2009; Liberman L. D. et al., 2015), rat (Bauer et al., 2007), guinea pig (Lin et al., 2011; Shi et al., 2013; Hickman et al., 2020), chinchilla (Hickox et al., 2017), gerbil (Gleich et al., 2016), rhesus monkey (Valero et al., 2017), and human (Viana et al., 2015; Wu et al., 2020). This primary neural degeneration has been observed in response to continuous noise exposures (Kujawa and Liberman, 2009), impulse noise exposures in chinchillas (Hickman et al., 2018), certain ototoxic drugs in humans and mice (Hinojosa and Lerner, 1987; Ruan et al., 2014) and simple aging of the ear in mice, gerbils, and humans (Sergeyenko et al., 2013; Gleich et al., 2016; Wu et al., 2020). In humans, it may be exacerbated by conditions such as Ménière’s disease (Nadol, 1988), and in mouse it can be brought on by chronic conductive hearing loss (Liberman M. C. et al., 2015). Noise-induced synaptic loss can be dramatic, e.g., as much as 50% in mouse (Kujawa and Liberman, 2009), even in cases where there has been no loss of hair cells, and no permanent threshold elevations. In rhesus macaques it can be even greater, with peak synapse losses averaging 75% in cases where the noise damage is severe enough to cause massive OHC loss and thus a significant permanent threshold shift (Valero et al., 2017). In the normal-aging human, the loss of ANF peripheral axons, which can occur subsequent to loss of the synaptic terminals in the organ of Corti, can also be dramatic, e.g., as great as 70% in aged individuals despite survival of virtually all the IHCs (Wu et al., 2019).

The data from older humans, gerbils and mice show that, at least in the “normal-aging” ear, synaptopathy is, to some extent, irreversible (Sergeyenko et al., 2013; Gleich et al., 2016; Wu et al., 2019), although there may be an ongoing balance between synaptic degeneration and regeneration. Likewise, in the noise-exposed rhesus ear, massive IHC synaptic loss has been observed as long as 8 months post exposure (Valero et al., 2017), thus whatever balance exists between ongoing degeneration and regeneration, it must bias toward degeneration.

**FIGURE 11:**
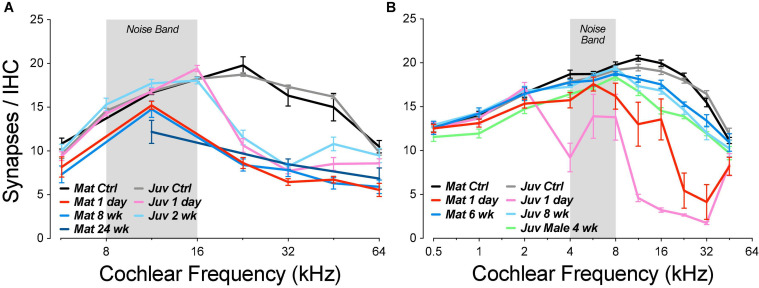
Noise-induced synaptic loss is effectively irreversible in CBA/CaJ mouse **(A)**, whereas there is significant post-exposure recovery in both juvenile and mature guinea pigs **(B)**. Data in **(A)** are replotted from prior publications in which mice were exposed for 2 h at either 8 weeks (juvenile) or 16 weeks (mature) to the octave-band noise at either 98 or 100 dB, respectively (Kujawa and Liberman, 2009; Fernandez et al., 2015; Liberman L. D. et al., 2015). Data from juvenile guinea pigs are from a prior report (Hickman et al., 2020). Data from mature guinea pigs are from the present report.

Guinea pig studies from the Wang laboratory were the first to suggest, based on ribbon counts, that there might be significant post-exposure regeneration of noise-damaged synapses, by 1 month post exposure (Shi et al., 2013). In prior mouse work, we studied the development and possible recovery from noise-induced synaptopathy over an extended time course. We used CBA/CaJ mice, since this inbred strain shows minimal age-related threshold shift until late in life (Zheng et al., 1999; Sergeyenko et al., 2013). We varied age-at-exposure, since mice show larger noise-induced threshold shifts (and hair cell damage) when exposed as juveniles vs. adults (Henry, 1983; Kujawa and Liberman, 2006), and *in vitro* studies show a reduction of cochlear neural regeneration with increasing age after drug-induced synaptic destruction (Green et al., 2012). Specifically, we studied (1) animals exposed at 6–8 wks and then followed from 0 h to 16 months post exposure (Jensen et al., 2015; Liberman L. D. et al., 2015) and (2) animals exposed at 16 weeks and followed for up to 2 years post exposure (Kujawa and Liberman, 2009; Fernandez et al., 2015). Neither group showed appreciable recovery of the noise-induced synaptic loss (Figure 11A). The synaptopathy was fully manifest immediately after the termination of the 2-h noise exposure (Liberman L. D. et al., 2015). There was no recovery of synaptic counts at longer post-exposure times, and, at the longest post-exposure survival times, the fractional loss of spiral ganglion cells, the cell bodies of the ANFs, closely approaches the fractional loss of synapses observed immediately post exposure (Kujawa and Liberman, 2009). The simplest interpretation of this close correspondence is that there is no ongoing regenerative process in this mouse model.

In contrast, other mouse studies (Shi et al., 2015; Kaur et al., 2019) have reported significant post-exposure synaptic recovery in C57BL/6, an inbred strain often used because of the availability targeted genetic manipulations or because wildtype animals show an early-onset, age-related hearing loss (Henry and Chole, 1980). However, the ears in these C57BL/6 synaptopathy studies were exposed as juveniles (5–6 weeks), as were the guinea pigs in the first report of synaptic regeneration (Shi et al., 2013) and in our prior study (Figure 11B; Hickman et al., 2020). Intriguingly, a third study of noise-induced synaptopathy in C57BL/6 mice reports almost complete recovery of synaptic counts after a noise exposure at 6 weeks of age, but virtually no recovery in mice exposed once at 6 weeks and then again at 10 weeks (Luo et al., 2020). Although the authors attribute the difference to the effects of repeated exposure, the confound of age-at-exposure was unresolved.

Uncovering the reason(s) for the differences in post-exposure synaptic recovery is important to our understanding of the mechanisms underlying the generation and progression of cochlear synaptopathy, and to the possible development of therapeutic approaches to prevent or reverse it in humans (e.g., Suzuki et al., 2016). Thus, a major aim of the present study was to compare synaptic regeneration in juvenile vs. mature animals. The present results suggest that differences in regenerative capacity are not directly attributable to a difference in age-at-exposure.

### Degeneration and Regeneration vs. Down- and Up-Regulation of Synaptic Proteins

Although examining the role of age does not exhaust the list of possible reasons for the discrepant outcomes *re* post-exposure synaptic recovery, it strengthens the suggestion that there are important interspecies or interstrain differences in the inherent regenerative capacity of cochlear neurons, even in adult ears. However, there is a fundamental confound in the interpretation of confocal images that also needs to be considered. Disappearance and reappearance of immunostaining, e.g., for pre- and post-synaptic puncta, is not unambiguous evidence for degeneration and regeneration of synapses or synaptic terminals. As illustrated in the high-power insets to Figure 6, careful examination of noise-exposed tissue commonly reveals extremely faint synaptic puncta that would be missed at lower power or with immunostaining protocols that produce lower signal-to-noise ratios. These images suggest downregulation of GluA2 protein expression at many synapses, rather than a frank loss of post-synaptic terminals. Such an inference is consistent with prior reports, using totally different methodology, suggesting that glutamate receptors at the ANF/IHC synapse are transiently internalized during an acoustic overexposure (Chen et al., 2007). The further observation that orphan ribbons are rare both in controls and by 4–6 weeks post exposure (Figure 7C; Hickman et al., 2020) suggests that many orphan ribbons at 1 day post exposure could reflect intact synaptic contacts that express extremely low numbers of glutamate receptors. The same caveat applies to the ribbon counts. CtBP2 expression is reduced post exposure such that mean ribbon volume falls even outside the main synaptopathic zone (Figure 10B). Thus, within the damage focus, CtBP2 expression in smaller ribbons could fall below the threshold for detection, depending entirely on the robustness of the immunostaining protocols.

Thus, the reduction and subsequent recovery of synaptic counts from ≤1 day to several weeks following exposure, *per se*, is not evidence for degeneration/regeneration of synaptic terminals and true synaptogenesis. There is a fundamental distinction between a scenario where terminals die back, resprout and reform synapses with the IHCs vs. an alternate scenario in which there is simply down- and subsequent up-regulation of synaptic proteins. This distinction is critical in the context of designing therapies for cochlear synaptopathy. To address this inherent limitation of confocal imaging, we supplemented our ribbon counts with (1) analysis of the pairing between pre- and post-synaptic puncta, and (2) a mapping procedure to reveal the spatial distribution of these orphaned and paired puncta around the IHC soma. These analyses clearly showed the synaptic machinery to be dynamic, even in the fully developed ear. In both juvenile and mature ears, ribbons and synaptic complexes must either migrate to, or regenerate at, locations quite distant from their normal mature positions lining the basolateral pole of the IHC (Figure 8).

**FIGURE 12:**
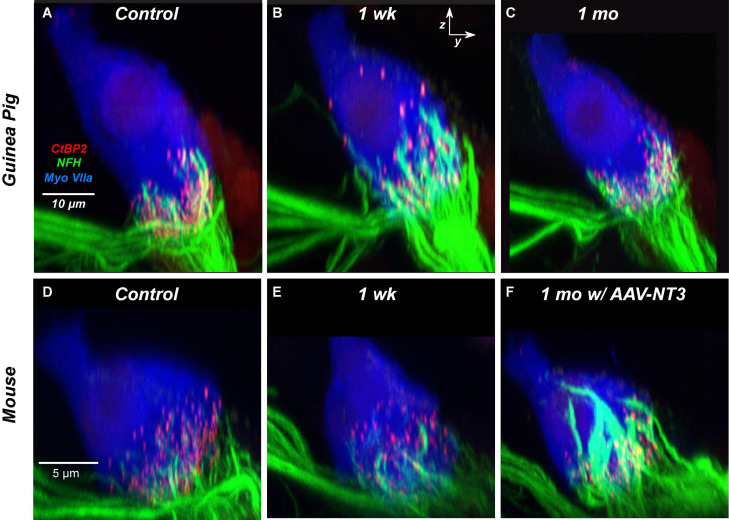
The neurite extension seen spontaneously in the noise-exposed guinea pig **(B)** has a similar morphology to the neurite extension seen in mouse ears treated with virally mediated NT3 overexpression, both exposed **(F)** and unexposed (not shown). Panels **(A–C)** are from the 22.6 kHz region and are taken from a prior study of noise-induced synaptopathy in juvenile guinea pigs (Hickman et al., 2020). Panels **(D–F)** are from the 32 kHz region and are taken from a prior study of noise-induced synaptopathy in CBA/CaJ mice pre-treated with AAV virus particles carrying an NT3 gene sequence (Hashimoto et al., 2019). Panels **(A,D)** are from unexposed controls; panels **(B,C,E,F)** are from exposed animals at the survival times indicated. All images are *zy* projections of confocal z-stacks, all very similar in *xy* field of view to the projections shown in Figures 6A,B.

To gain further insight into the nature of the recovery process occurring after synaptopathic noise, in our prior study of juvenile guinea pigs, we added a fourth immunomarker, i.e., for neurofilament, to assess the loss and/or recovery of ANF terminals in close apposition to the pre- and post-synaptic puncta at various positions around the IHC basolateral surface. As illustrated by the confocal projections in Figure 12, many of the “displaced” synaptic puncta at positions close to the IHC cuticular plate are close to NF-positive terminals that extend much higher up the IHC membrane than in the normal ear. Furthermore, these putative ANF extensions contain significantly more neurofilament immunoreactivity, a possible marker of outgrowing neurites. Such thickened and elongated ANF terminals do not appear spontaneously in the noise-exposed CBA/CaJ mouse (Figure 12E), which also shows minimal recovery of synaptic counts post exposure, but are present in great numbers in mice pre-treated with a virus that elicits overexpression of the neurotrophin NT3 in IHCs (Figure 12F) and enhances synaptic recovery post exposure (Hashimoto et al., 2019).

Neurotrophins such as NT3 and BDNF are key to neuronal survival in the adult inner ear (Stankovic et al., 2004). NT3, which is especially critical for ANFs, is expressed by neonatal IHCs and their surrounding supporting cells, and its expression levels fall steadily from apex to base and with increasing age (Sugawara et al., 2007). Endogenous neurotrophin expression in adulthood or post injury could be a key determinant of the degree of synaptic recovery after noise, and there could be important differences between guinea pig and mouse, and/or between CBA/CaJ and C57BL/6 mice. If the slow age-related NT3 decline seen in CBA/CaJ is recapitulated in humans, it could be a major driver of the slow age-related decline in ANF survival observed in the absence of significant IHC loss (Wu et al., 2019).

### Selective Low-SR Fiber Loss and the Modiolar/Pillar Gradients in Synaptic Morphology

In a prior study of juvenile guinea pigs studied 3–6 weeks after an identical acoustic overexposure to that used here, ANF responses of surviving ANFs were normal in threshold, tuning, and a variety of suprathreshold response measures (Furman et al., 2013). Sampling statistics suggested a selective loss of fibers with low SRs and high thresholds. Since these low-SR fibers are particularly important in extending the dynamic range of the auditory periphery and in maintaining AN response to signals in the presence of masking noise (Costalupes et al., 1984), the idea that acoustic trauma selectively eliminates the low-SR fibers would help explain why many with noise-induced hearing loss have particular problems understanding speech in difficult listening environments (Gordon-Salant and Cole, 2016).

In prior studies, the morphological analyses of synaptopathy failed to find damage patterns consistent with the selective loss of low-SR fibers (Furman et al., 2013; Hickman et al., 2020). Single-fiber labeling studies have shown that low-SR fibers preferentially contact the modiolar side of the IHCs, where they form synapses with larger pre-synaptic ribbons than those opposite the high-SR fibers on the pillar side of the IHC (Merchan-Perez and Liberman, 1996; Tsuji and Liberman, 1997). Thus, the distinction between modiolar- and pillar-side synapses as well as the normal ribbon-size gradient in confocal *zy* projections (e.g., Figures 6C,E) are robust morphological markers of this important functional distinction among ANFs. Prior studies reported reduced synaptic counts, but restricted their spatial analyses of modiolar-pillar distinctions to consideration of *ribbon* counts and sizes. With respect to these pre-synaptic elements, prior studies agree that ribbons become larger in the synaptopathic region, as shown here in Figure 10 for both modiolar -and pillar-side ribbons, however, there was no compelling evidence that the noise-induced loss was greater on the modiolar side. The spatial analysis of synaptic puncta before and after exposure presented here, and the preferential reduction in the numbers of modiolar-side synapses it revealed (Figure 9) represents the most definitive morphological corroboration to date of the idea that noise exposure chiefly targets ANFs with low-SRs and high thresholds. One key insight appears to be that the post-synaptic disruption is more extensive than the pre-synaptic disruption. The second is the revelation that synapses are migrating and reforming in first few weeks post exposure. This can explain why the initial single-fiber study failed to find clear-cut morphological evidence for a polarization of damage around the IHC circumference, given that all the histopathology was performed at least 3 weeks post exposure.

Other morphological characteristics of the low-SR fibers include their smaller caliber and lower mitochondrial content (Liberman, 1980); the latter may contribute to their vulnerability to noise, given that the noise-induced synaptopathy is thought to arise from glutamate excitotoxicity and the excess Ca^2+^ entry it allows (Hu et al., 2020), and mitochondria are a key source of intracellular Ca^2+^ buffering (Szydlowska and Tymianski, 2010). The lack of complete morphological recovery of the architecture of surviving synapses, as seen in the confocal data extracted from thousands of synaptic puncta (Figure 10B), suggests that more careful physiological analysis of larger numbers of ANF might reveal subtler pathophysiologies in the neurons surviving after these severe, but ultimately reversible noise-induced threshold shifts.

## Data Availability Statement

The raw data supporting the conclusions of this article will be made available by the authors, without undue reservation.

## Ethics Statement

The animal study was reviewed and approved by IACUC of the Massachusetts Eye and Ear.

## Author Contributions

TH and MCL designed the experiments. TH and KH acquired the physiological data. LL designed and performed the immunostaining protocols. TH, KH, and MCL acquired the confocal data. All authors participated in the data analysis. MCL and TH wrote the manuscript, and all other authors edited it.

## Conflict of Interest

The authors declare that the research was conducted in the absence of any commercial or financial relationships that could be construed as a potential conflict of interest.

## Publisher’s Note

All claims expressed in this article are solely those of the authors and do not necessarily represent those of their affiliated organizations, or those of the publisher, the editors and the reviewers. Any product that may be evaluated in this article, or claim that may be made by its manufacturer, is not guaranteed or endorsed by the publisher.
